# Model Analysis and Experimental Investigation of Soft Pneumatic Manipulator for Fruit Grasping

**DOI:** 10.3390/s22124532

**Published:** 2022-06-15

**Authors:** Yinlong Zhu, Kai Feng, Chao Hua, Xu Wang, Zhiqiang Hu, Huaming Wang, Haijun Su

**Affiliations:** 1 College of Mechanical and Electronic Engineering, Nanjing Forestry University, Nanjing 210037, China; ylzhu@njfu.edu.cn (Y.Z.); fkai@njfu.edu.cn (K.F.); m18252076750@163.com (C.H.); 2 State Key Laboratory of Robotics, Shenyang Institute of Automation, Chinese Academy of Sciences, Shenyang 110169, China; hzq@sia.cn; 3 College of Mechanical and Electrical Engineering, Nanjing University of Aeronautics and Astronautics, Nanjing 210016, China; hmwang@nuaa.edu.cn; 4 Department of Mechanical and Aerospace Engineering, Ohio State University, Columbus, OH 43210, USA; su.298@osu.edu

**Keywords:** soft manipulator, pneumatics, soft robot, fruit grasping

## Abstract

With the superior ductility and flexibility brought by compliant bodies, soft manipulators provide a nondestructive manner to grasp delicate objects, which has been developing gradually as a rising focus of soft robots. However, the unexpected phenomenon caused by environmental effects, leading to high internal nonlinearity and unpredictable deformation, makes it challenging to design, model, and control soft manipulators. In this paper, we designed a soft pneumatically actuated manipulator consisting of four soft actuators, as well as a flange, and investigated the influence of structural parameters on the output characteristics of the manipulator through finite element analysis (FEA). To enhance the bending deformation of the soft actuator, annular rings were employed on the soft actuator. A mathematical model for the bending deformation of air cavities was established to explore the relationship between the driving pressure and the bending angle based on the Yeoh strain energy function. Moreover, an end-output force model was established to depict the variation of the force output with the bending angle of the soft actuator, which was then experimentally validated by adopting the manufactured manipulator. The soft actuator studied in this paper can bend from 0° to 110° under an applied pressure of 0–60 kPa, and the maximum grasping load of the soft manipulator is 5.8 N. Finally, practical tests were conducted to assess the adaptability of the soft manipulator when grasping delicate fruits, such as apples, pears, tomatoes, and mangoes, demonstrating its broad application prospects in nondestructive fruit harvesting.

## 1. Introduction

Robotic fruit harvesting has been a research hotspot for over 40 years, as it can tremendously reduce the reliance of fruit growers on a largely seasonal and often untrained labor force [1,2,3]. However, nowadays, most of these robots still use traditional rigid manipulators, which show poor adaptability in grasping fruits of different sizes and shapes [4,5]. Particularly, when rigid manipulators are applied to grasp delicate fruits [6], such as tomatoes, raspberries, and strawberries, their stiff structures, producing point forces on the target may cause contact damage to the object surface [7].

The emergence of soft actuators provides new ideas and methods for fruit harvesting. The actuating methods mainly include pneumatic driving [8], tendon driving [9], and intelligent material driving, such as shape memory [10], piezoelectric [11], electroactive polymer [12,13], and magnetostrictive materials [14]. Compared with rigid manipulators, soft manipulators have diverse advantages, including low weight, safety, and extremely strong environmental adaptability due to their infinite degrees of freedom [15,16]. Soft manipulators provide an alternative to traditional rigid manipulators to solve the problem of complex structures and poor interaction [17,18,19]. Pneumatic actuation is commonly used for soft manipulators, in which compressed air can be stored and dispensed at precise pressure levels. When grasping targets, pressurized air is utilized to cause the inflation/deflation of the inner cavities embedded in the actuators, thus resulting in the bending deformation of actuators. Hao et al. [20] designed a soft actuator based on a pneumatic drive, which can change its length according to the shape of the object to achieve a stable grasp. Soft grippers have recently made great progress, mainly due to the development of materials and stretchable electronics [21]. Soft grippers are an integral part of the robotics systems used in smart farming technologies [22]. Various soft grippers have been developed recently. Wehner et al. [23] used silicone rubber materials to produce a pneumatic network-based actuator, which has the properties of large deformation, good flexibility, and good interactivity. She et al. [24] designed a soft actuator with built-in sensors, which can reflect the working status of the gripper in real time, and has the properties of flexible movement and good interactivity. Brown’s team [25] designed a new type of soft robot based on particle plugging, which is composed of soft materials and granular coffee beans. By applying negative pressure, it can adjust the stiffness of the overall structure, adapt to the shape of objects when grasping them, and improve the anti-interference ability by increasing the stiffness. Wang [26] presented a circular gripper with soft air chambers to hold various food-shaped objects, with which flexible motions could be achieved. Jinho [27] proposed a promising and convenient method to sense the surface information of a soft robotic arm through stretchable silicones and one-dimensional carbon materials.

These pneumatic soft actuators are often built with high-deformable and elastic materials, such as silicone polymers, elastrators, and rubbers, which exhibit nonlinear stress–strain properties, making it difficult to model soft actuators and achieve precise control [28,29]. Implementing the motion of soft materials has become a key problem in designing soft manipulators [30,31]. Although soft actuators have potential applications in various fields, the basic principles for the response model of deformation and force output under various air pressure conditions require further development. Soft bending actuators have drawn significant attention recently, but limited modeling work has been conducted. Due to the nonlinear response and complex geometry, it is complicated to acquire a systematic understanding of the relationship between actuator geometry and its performance.

In this paper, a soft manipulator consisting of four pneumatic actuators was designed and manufactured, and the optimal structural parameters were investigated through simulation analysis. Based on the Yeoh strain energy function and the assumption of constant curvature, a bending characteristic model and an end-output force model of the soft actuator were established. An experimental platform was established to test the bending angle and output force of the soft actuator. Through the grasping experiment, the proposed soft manipulator can realize the nondestructive grasping of various kinds of fruits.

## 2. Design and Fabrication of Soft Manipulator

### 2.1. Structure Design

Soft pneumatic actuators (SPAs) usually use the local strain difference to generate bending degrees of freedom. In other words, under the same air pressure, the strain of different stiffness material layers is different, and uneven displacements will occur between the layers. Driven by air, the soft actuator bends towards the constraint layer [32].

**Figure 1:**
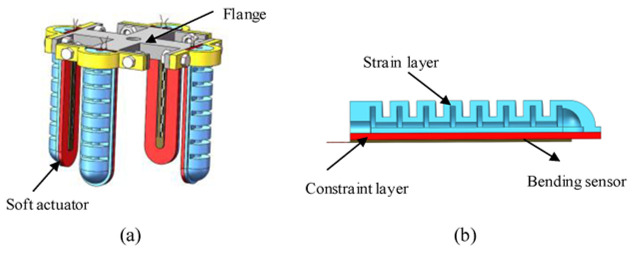
(**a**) Structure diagram of soft manipulator; (**b**) internal structure diagram of soft actuator.

Based on the above principle, the soft manipulator consists of four soft actuators and a flange, as shown in Figure 1. Each actuator consists of a strain layer and a constraint layer. The bending sensor is embedded in the surface of the constraint layer to measure the bending angle. When inflated with positive pressure, the volume of the chamber in the strain layer increases, and then the actuator bends toward the constraint layer; when the pressure is negative, the contraction rate of the strain layer is greater than the contraction rate of the constraint layer, and the actuator bends toward the strain layer.

### 2.2. Fabrication Process

**Figure 2:**
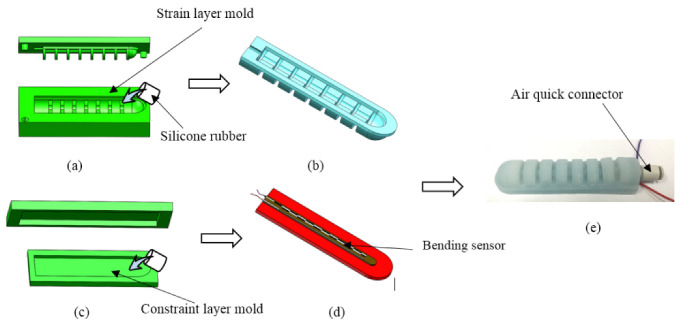
Soft actuator preparation flow chart. (**a**) Preparation of strain layer with mold. (**b**) Prepared strain layer. (**c**) Preparation of limiting layer. (**d**) Attachment of the bending sensor. (**e**) The assembled soft manipulator.

**Figure 3:**
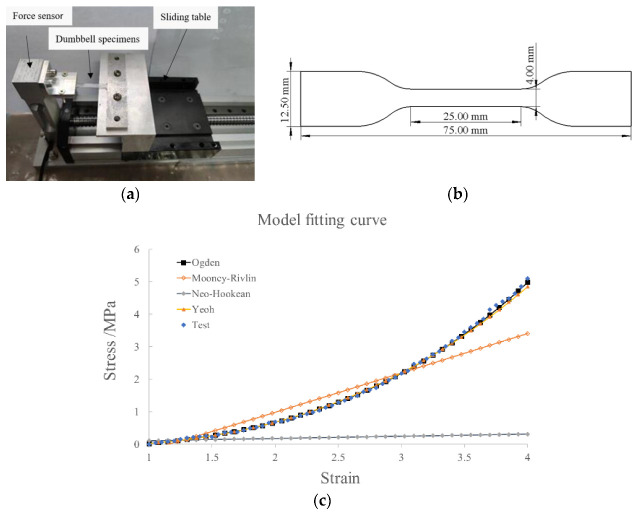
(**a**) A uniaxial tensile test was performed; (**b**) the averaged nominal stress–strain curve of silicone rubber and model fitting curve; (**c**) size information of the dumbbell-shaped specimens.

In this paper, the soft actuator is fabricated by lamination casting. All of the molds are fabricated by 3D printers. The mold is composed of a strain layer mold and a limit layer mold, as shown in Figure 2. The preparation process is listed as follows:

(1) The two parts of silicone rubber (Dragon Skin 20 by Smooth-ON company, Macungie, PA, USA) A and B were mixed at a ratio of 1:1 by a mixer.
(2) The uncured silicone was then poured into the molds for the strain layer and constraint layer. In order to eliminate bubbles, the molds were degassed in a vacuum chamber (see Figure 2a,c);
(3) After 5 h at room temperature, the strain layer (see Figure 2b) and constraint layer (see Figure 2d) were removed from the molds. Then, the bending sensor was bonded to the constraint layer with uncured silicone.
(4) Finally, the strain layer and constraint layer were assembled with an air quick connector inserted in the air hole (see Figure 2e).

## 3. Finite Element Analysis of Soft Actuator

The structural parameters of the designed soft actuator mainly include the chamber thickness, the number of chambers, the gap between the chambers, and the thickness of the constraint layer. The ABAQUS finite element simulation software was used to analyze the effect of the structural parameters on the bending properties and obtain the optimal structural parameters.

To illustrate the material characteristic of silicone rubber, we performed a uniaxial tensile test, as shown in Figure 3a. Three specimens were stretched at 20 mm/min, and then the averaged experimental data were inputted into the material evaluation tool of ABAQUS. Four strain energy form functions (Mooney–Rivlin, Ogden, Neo–Hookean, and Yeoh) were fitted with the experimental data separately to determine the material parameters. As shown in Figure 3c, the Yeoh form (with C10 = 0.11 and C20 = 0.02) is consistent with the experimental data well. The sizes of the dumbbell-shaped specimens are illustrated in Figure 3b.

In the simulation process, the fluid cavity model was adopted to conduct the inflation simulation, and the left end of the soft actuator was completely constrained. Due to the large deformation, the geometrically nonlinear switch needed to be turned on in the analysis step. In the setting of the mesh unit type, a tetrahedral mesh was selected with a quadratic geometric order. Considering the incompressibility of the silicon material, a hybrid unit type was adopted to mesh division.

### 3.1. Effect of Cavity Wall Thickness on Bending Angle

**Figure 4:**
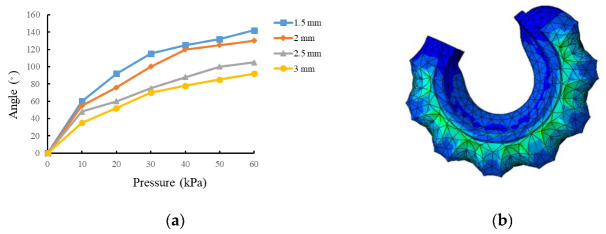
Effect of cavity wall thickness on bending properties. (**a**) Simulation results of different cavity wall thickness; (**b**) BENDING simulation diagram with wall thickness of 1.5 mm at 40 kPa.

The simulation analysis of soft actuators with cavity wall thicknesses of 1.5 mm, 2.0 mm, 2.5 mm, and 3.0 mm was carried out, in which the number of chambers was 8, the gap between the chambers was 3 mm, and the thickness of the constraint layer was 2.5 mm. The simulation results are shown in Figure 4a. It can be seen that the larger thickness of the cavity wall leads to a smaller bending angle under the same air pressure, and the bending angle gradually increases with the increase in air pressure. The simulation diagram of the soft actuator with a wall thickness of 1.5 mm inflated by air pressure of 40 kPa is shown in Figure 4b. The radial expansion of the soft actuator is clear when the thickness is too thin. This phenomenon also illustrates the large stress distribution at the junction of the constraint layer and strain layer, which could lead to the bursting of the soft actuator. However, a thickness of 3 mm cannot produce an appreciable bending angle. Considering all the factors, the thickness of 2.5 mm was chosen in the process of fabricating the soft actuator prototype.

### 3.2. Effect of Chamber Clearance on Bending Properties

The simulation analysis was carried out on the soft actuators with chamber gaps of 1 mm, 3 mm, and 5 mm. The cavity wall thickness was 2.5 mm, the number of chambers was 8, and the thickness of the constraint layer was 2.5 mm.

**Figure 5:**
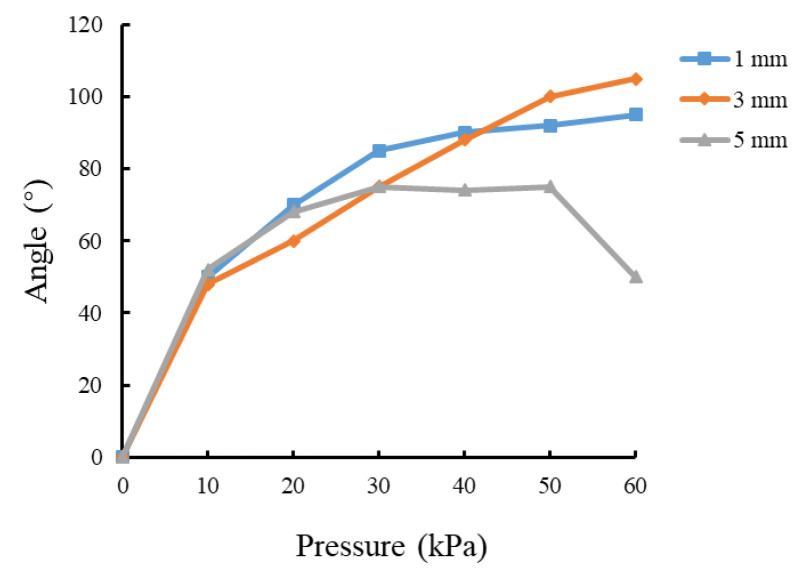
Effect of chamber clearance on bending properties.

The simulation results of the effect of the chamber gap on the soft actuator bending properties are shown in Figure 5. When the gap is 1 mm and the air pressure is higher than 30 kPa, the deformation between adjacent chambers will be disturbed due to the small gap between the chambers, and large deformation of the constraint layer results in little change in the bending angle. When the clearance is 5 mm and the air pressure varies from 20 to 50 kPa, the effect of compressed air mainly changes the volume of the air bag instead of acting on the constraint layer; thus, the bending angle changes slightly. When the air pressure is over 50 kPa, the bending angle decreases rapidly due to the failure of the soft actuator resulting from the excessive inflation of air bags. Therefore, a soft actuator structure with a 3 mm gap was chosen in this study.

### 3.3. Effect of Constraint Layer Thickness on Bending Properties

The simulation analysis was carried out on the actuator with constraint layer thicknesses of 1.5 mm, 2.0 mm, 2.5 mm, and 3.0 mm. The cavity wall thickness was 2.5 mm, the number of chambers was 8, and the gap between chambers was 3 mm.

**Figure 6:**
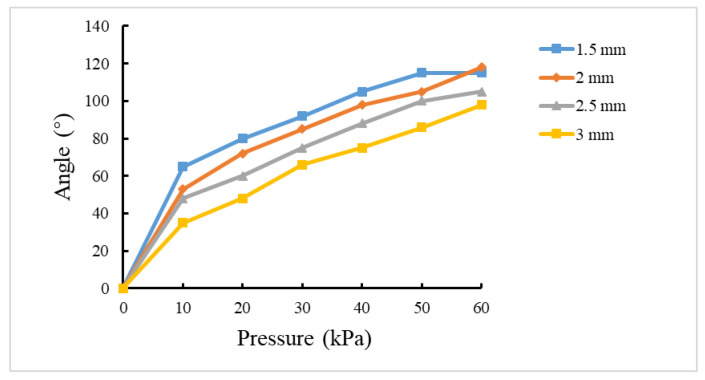
Effect of constraint layer thickness on bending properties.

As shown in Figure 6, the greater the constraint layer’s thickness, the smaller the bending angle. If the thickness of the constraint layer is too thin, such as 1.5 mm, the bending angle could decrease when inflated by air pressure over 50 kPa. This phenomenon can be explained as excessive deformation of the constraint layer will decrease the bending angle.

The soft actuator’s structural parameters must not only meet the requirement of the bending angle but also avoid the failure of the soft actuator due to radial expansion and other reasons. Based on the theoretical analysis, the optimal structural parameters could be obtained as follows: the wall thickness of the cavity was 2.5 mm, the number of chambers was 8, and the chamber gap was 3 mm.

**Figure 7:**
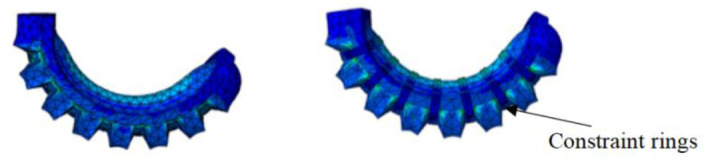
Comparison diagram of finite element simulation with and without constraint rings.

FEM simulation shows that the radial expansion of the soft actuator consumes strain potential energy and even results in the failure of the actuator, which will reduce the bending angle of the soft actuator. To reduce the radial expansion deformation, an annular structure could be adopted to limit the radial expansion. Figure 7 is a comparison of the radial expansion of the soft actuator with and without constraint rings. It can be easily seen that the bending angle increases when the annular ring is used. To significantly promote the output force of soft actuators, embedding and winding methods were introduced, which could enhance the strength of soft structures.

## 4. Modeling and Analysis of Soft Actuator

The soft manipulator is made of silicone material, which is a kind of hyperelastic material, and its mechanical properties are nonlinear. In order to analyze its mechanical properties, it is assumed that the hyperelastic materials are incompressible and perform isotropy without deformation. Based on the above assumptions, the constitutive model of silicone is expressed by strain energy density, and the constitutive models usually used include Mooney–Rivlin, Ogden, the Yeoh model, etc.

The Yeoh model has good adaptability within 100% deformation, and it is the preferred constitutive model for analyzing the deformation of silicone. The internal strain energy of the material is used to describe the mechanical properties of silicone materials [33]. The strain energy density function *W* could be expressed by the invariants of strain tensors *I*_1_, *I*_2_, and *I*_3_. The strain potential energy of the Yeoh form [34] could be written as:

(1) $$W=C_{10}(I_{1}-3)+C_{20}{\left(I_{1}-3\right)}^{2}$$

(2) $$\mathrm{where}\;I_{1}=\lambda_{1}^{2}+\lambda_{2}^{2}+\lambda_{3}^{2},\;I_{2}=\lambda_{1}^{2}\lambda_{2}^{2}+\lambda_{1}^{2}\lambda_{3}^{2}+\lambda_{2}^{2}\lambda_{3}^{2},\;I_{3}=\lambda_{1}^{2}\lambda_{2}^{2}\lambda_{3}^{2}$$

in which *λ*_i_ is the stretch ratio in the ith direction. The silicone material is assumed to be incompressible, thus $I_{3}=1$.

The true principal stress (also called Cauchy stress) $\sigma_{i}$ can be obtained from the partial derivative of the principal extension ratio by the strain energy function:

(3) $$\sigma_{i}=\lambda_{i}\frac{\partial W}{\partial \lambda_{i}}-p=2\lambda_{i}^{2}[C_{10}+2C_{20}(I_{1}-3)]-p,\;(i=1,\;2,\;3)$$

### 4.1. Relationship between Driving Pressure and Bending Angle

**Figure 8:**
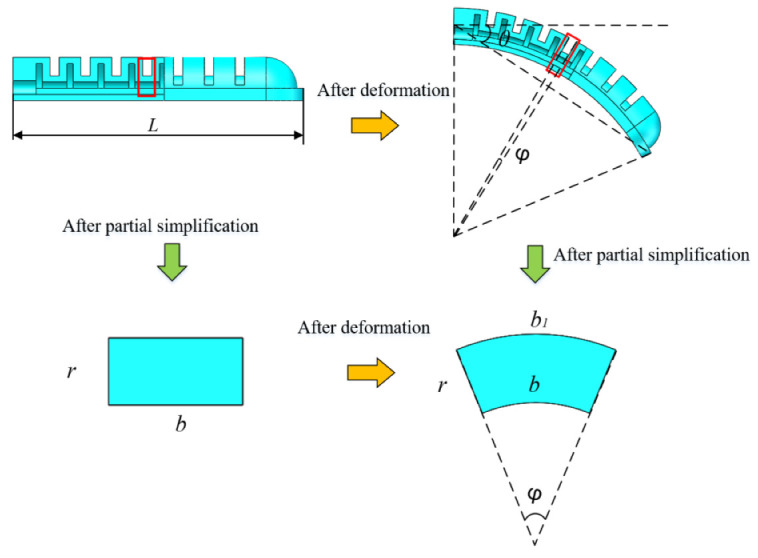
Simplified schematic diagram of cavity structure deformation.

Considering the geometric deformation of the soft actuator is complicated to simulate, it is assumed to be a constant curvature model, and there is no deformation in the circumferential direction. Thus, the cavity structure between the two adjacent chambers was selected for analysis. The detailed structure of the soft actuator is shown in Figure 8, and the item *P* is the compressed air pressure, *θ* is the bending angle of the soft actuator, *b* is the cavity length, *t* is the wall thickness, *L* is the length of the soft actuator, and *φ* is the bending angle of one clearance structure, and *t*_0_ is the original thickness of the wall.

The relationship between the overall bending angle *θ* of the soft actuator and the cavity bending angle *φ* can be written as:

(4) θ=Lφ/(2b)

Since it is assumed that there is no deformation in the circumferential direction, according to the geometric relationship, we have

(5) $$b_{1}=b+r\varphi$$

(6) $$\lambda_{1}=b_{1}/b=(b+r\varphi )/b,\;\lambda_{2}=1$$

(7) $$\lambda_{3}=1/\lambda_{1}$$

where *λ*_1_, *λ*_2_, and *λ*_3_ denote the stretch ratio in the axial direction, circumferential direction, and the direction of wall thickness, respectively.

**Figure 9:**
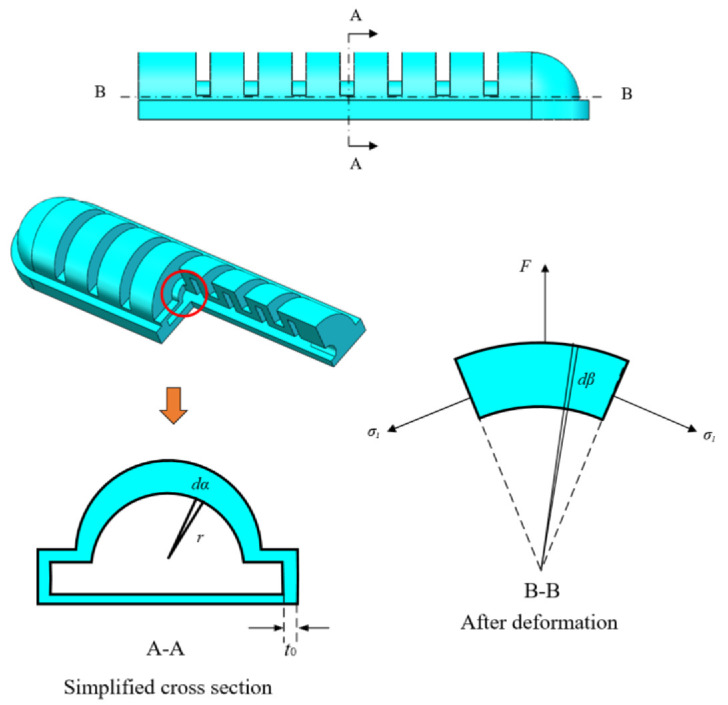
Cavity bending force balance diagram.

The force balance diagram of the bending cavity is shown in Figure 9, and the net force *F* resulting from compressed air acting on the upper surface of the cavity is:

(8) $$F=\int_{0}^{\pi }\int_{0}^{\varphi }Pr\left(\frac{b}{\varphi }+r\sin\alpha \right)\sin\alpha \cos\left(\frac{\varphi }{2}-\beta \right)d\alpha d\beta$$

The force balance equation in the vertical direction is:

(9) $$F=\sigma_{1}\sin\frac{\varphi }{2}\times (\pi {\left(r+\lambda_{3}t_{0}\right)}^{2}-\pi r^{2})$$

Combining Equations (8) and (9) above, the balance equation can be obtained as:

(10) $$Pr\sin\frac{\varphi }{2}\times (\frac{4b}{\varphi }+r\pi )=\sigma_{1}\sin\frac{\varphi }{2}\times \pi ({\left(r+\lambda_{3}t_{0}\right)}^{2}-\pi r^{2})$$

Considering there is no deformation in the circumferential direction and *σ*_2_ = 0, the hydrostatic pressure p could be determined. Thus, the axial principal stress *σ*_1_ is:

(11) $$\sigma_{1}=2(\lambda_{1}^{2}-\lambda_{2}^{2})[C_{10}+2C_{20}(I_{1}-3)]$$

Substituting Equation (11) into the equilibrium Equation (10), it can be rewritten as

(12) $$Pr(\frac{4b}{\varphi }+r\pi )=2\pi (\lambda_{1}^{2}-\lambda_{2}^{2})[C_{10}+2C_{20}(I_{1}-3)]\times [{\left(r+\lambda_{1}^{-1}t_{0}\right)}^{2}-r^{2}]$$

**Figure 10:**
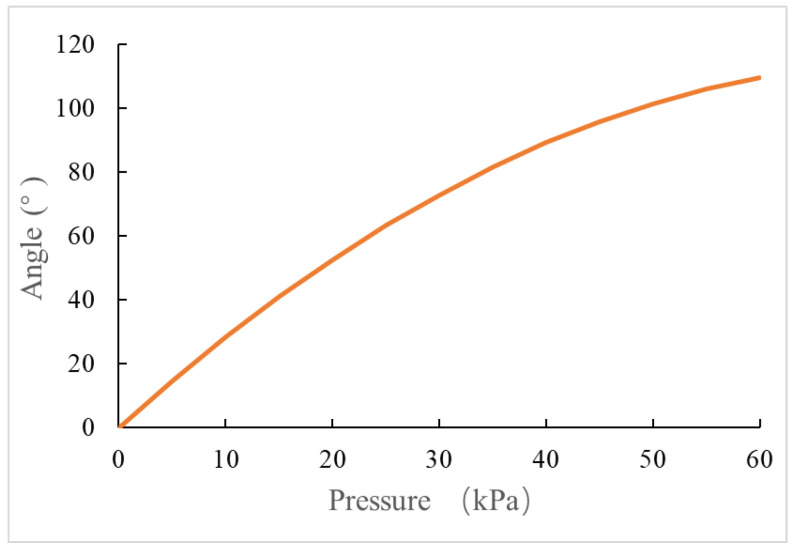
Relationship between bending angle and inflation pressure.

Equation (12) refers to the relationship between gas pressure *P* and cavity bending angle. Through Formula (4), the bending angle of the overall soft actuator can be obtained, as shown in Figure 10.

### 4.2. End-Output Force Model

According to the above model, the relationship between the pressure *P* and the bending angle *θ*_0_ is known. When the soft actuator bends to the maximum angle, the end-output force is 0. Therefore, the end-output force is only available during the bending process. In this paper, the large-deformation theoretical model of the flexible rod is used to depict the terminal output force.

**Figure 11:**
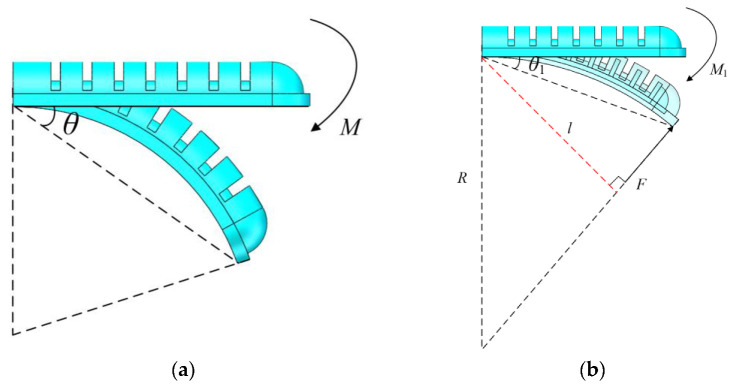
Flexible rod large-deformation equivalent model. (**a**) Free bending state of soft actuator. (**b**) After the soft actuator is bent to a free state, a reverse thrust is applied.

As shown in Figure 11, when inflated by compressed pressure *P*, which produces an equivalent moment *M* at the end, the angle *θ* can be given as

(13) $$\theta_{0}=ML/\left(2EI\right)$$

where *E* is elasticity modulus, *I* is inertia moment. The value of *E* is equal to the slope of stress–strain curve within *λ* = (1, 4) in Figure 3b, and *I* = 476.3 mm^4^ is the total actuator’s moment of inertia.

When the soft manipulator grasps an object, the soft actuator bends to angle *θ*_1_, the end-output force *F* acts vertically on the surface between the soft actuator and the grasped object. It can be equivalent to a moment *M*_1_ acting on the end of the soft actuator, which makes the soft actuator bend to angle *θ*_1_. The angle can be written as

(14) $$\theta_{1}=M_{1}L/\left(2EI\right)$$

Then, the end-output force of the soft actuator can be listed as

(15) $$F=\left(M-M_{1}\right)/l=\frac{M-M_{1}}{Rsin(2\theta_{1})}=\frac{4EI\theta_{1}(\theta_{0}-\theta_{1})}{Lsin(2\theta_{1})}$$

where *l* is the arm of force.

**Figure 12:**
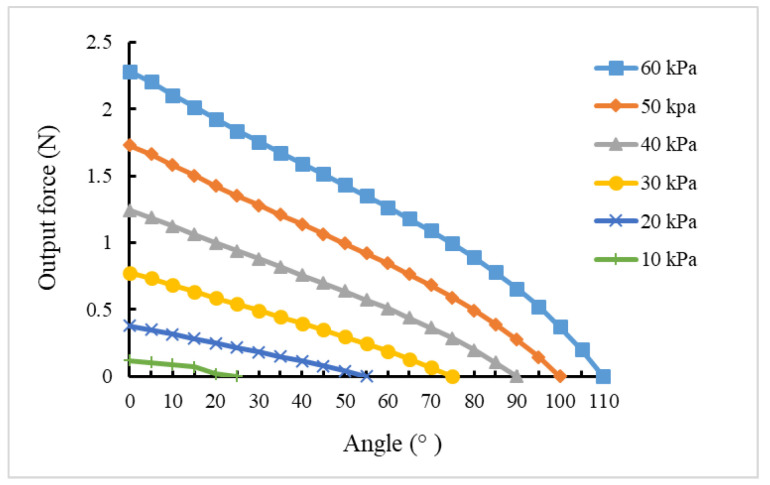
Relationship between the end-output force and the bending angle.

According to the output force model, the output force at each bending angle actuated by different pressure is shown in Figure 12.

## 5. Experimental Analysis of Soft Manipulator

### 5.1. Bending Angle Experiment

**Figure 13:**
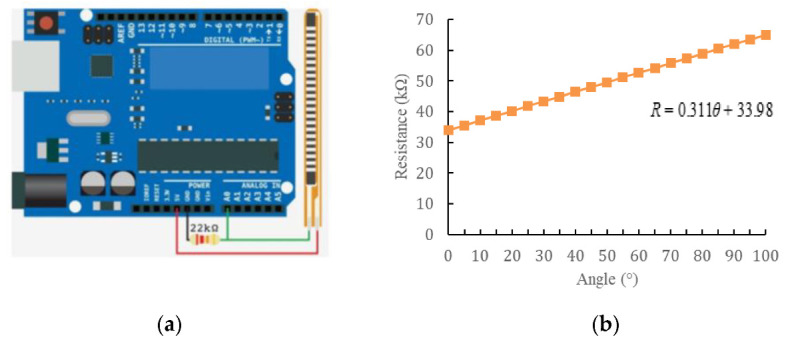
Flex Sensor 2.2. (**a**) Circuit diagram of bending sensor. (**b**) The relationship between the resistance value of the bending sensor and the bending angle.

In this experiment, Flex Sensor 2.2 was used to measure the bending angle of the soft manipulator. Figure 13a is its circuit design diagram. The circuit design of the bending sensor is essentially a voltage divider circuit, and its resistance value could be calculated according to Formula (16). Combining the relationship between resistance and angle, as shown in Figure 13b, the relationship between bending angle and voltage as shown in Formula (17) can be obtained.

(16) $$R_{i}=\left(5-u\right)\times 22/u$$

(17) θ=(5−u)×70.74/u−109.3

where *R*_i_ is the resistance value of the bending sensor, and *u* is the voltage assigned to the bending sensor.

**Figure 14:**
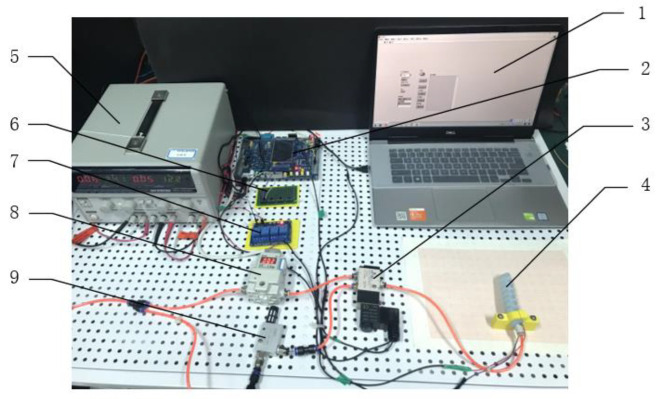
Experimental platform. 1. Host computer; 2. Stm32 MCU; 3. Solenoid valve; 4. Soft actuator; 5. DC power supply; 6. Triode amplifier circuit; 7. Relay module; 8. Proportional valve; 9. Vacuum generator.

The experimental platform for the soft actuator is shown in Figure 14. The Stm32 microcontroller was used to communicate with LabVIEW software to obtain the output air pressure of the proportional valve and the bending angle of the bending sensor in real time. The relay module controls the solenoid valve to change the flow direction of compressed air and the MCU outputs an analog voltage to proportional valves to adjust the value of air pressure. The flexible resistive sensor supplies the bending status.

**Figure 15:**
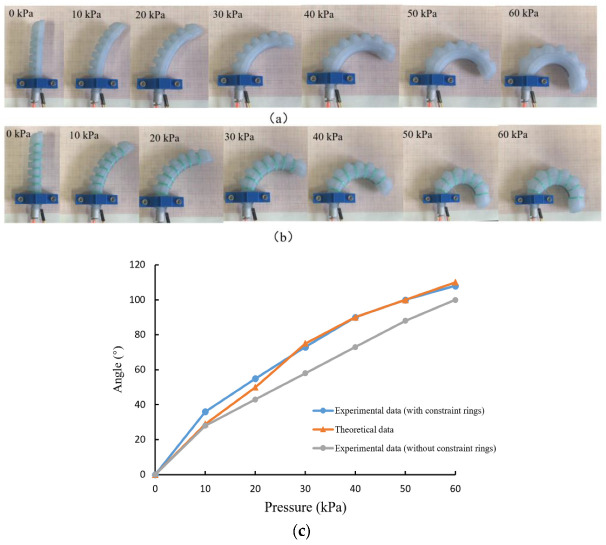
Results of bending experiments of soft actuators. (**a**) The bending angle of the soft actuator under different air pressure without constraint ring. (**b**) The bending angle of the soft actuator under different air pressure with constraint ring. (**c**) Comparison of experimental structure and theoretical results of soft actuator bending angle.

The bending experiment of the actuator driving under various air pressures was conducted, as shown in Figure 15. The comparison between the experimental results and theoretical analysis is shown in Figure 15c. For soft actuators with reinforced rings, the theoretical analysis results agree well with the experimental results, which can accurately predict the bending angles under certain pressure. However, when the air pressure is lower than 20 kPa, the error is relatively large, and the reasons may be as follow: (1) The assumption of constant curvature may result in deviation; (2) There is a certain error between the material parameters of the constitutive model and the actual mechanical behavior. Moreover, experimental results show that the soft actuator without constraint rings can achieve smaller bending deformation that verifies the FEM simulation results in Figure 4.

### 5.2. End-Output Force Experiment

**Figure 16:**
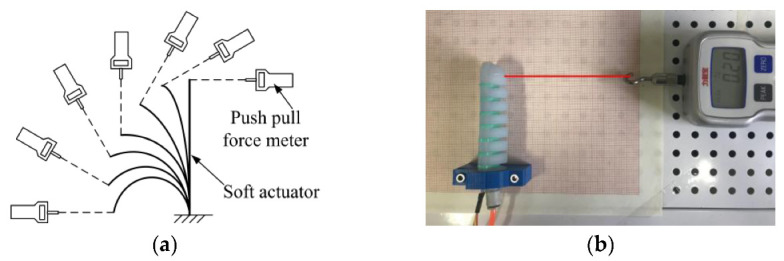
Output force experiment at the end of soft actuator. (**a**) Schematic diagram of the output force test at the end of the soft actuator. (**b**) Experimental diagram of the output force at the end of the soft actuator.

The output force at the end of the soft actuator reflects its load capacity, and the experiment program and device shown in Figure 16 were designed to measure the force.

The output force of the soft actuator with or without constraint rings under various air pressures was measured with a push–pull force meter at bending angles of 0°, 15°, 30°, 45°, 60°, 75°, and 90°. The experimental results are shown in Table 1 and Table 2. In the bending process of the soft actuator, it can be seen that the end-output force decreases with the increase in the bending angle. When the bending reaches the maximum angle, the end-output force is 0. At the same bending angle, the higher the inflation pressure, the greater the end-output force. As is well known, when the soft manipulator grabs the object, the appropriate air pressure should be selected according to the size of the grasped object and the size of the grasping force. Comparing Table 1 and Table 2, it can be seen that the end-output force of the actuator with the restriction ring is greater.

### 5.3. Grasping Experiment

**Figure 17:**
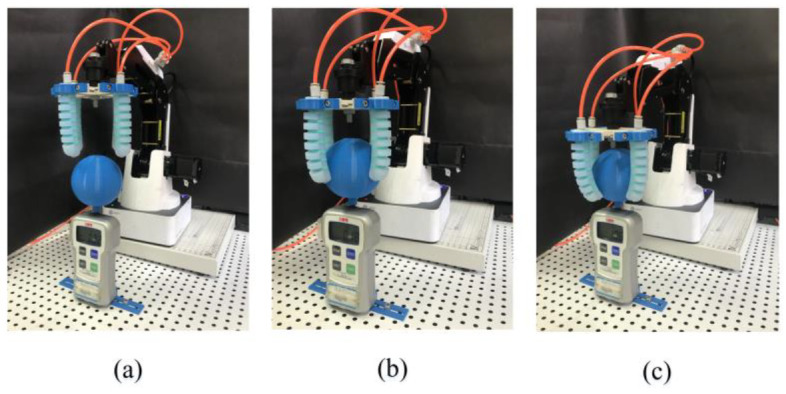
Experiment of fingertip grasping and envelope grasping. (**a**) Experimental diagram of grasping. (**b**) Experimental diagram of fingertip grasping (**c**) Experimental diagram of envelope grasping.

The pneumatic soft manipulator was fixed on the wrist of the DOBOT Magician robot, as shown in Figure 17. While the robot arm was moving upward, the soft manipulator would release the blue ball. The maximum pull force was measured using a force gauge (FGJ-50, Shimpo Company, Kyoto, Japan). Usually, the grasping manner includes enveloping grasping and fingertip grasping [35]. Table 3 shows the output force data of the soft manipulator under different air pressures with two grasping manners. It can be seen that the envelope grasping force is greater than the fingertip grasping force.

**Figure 18:**
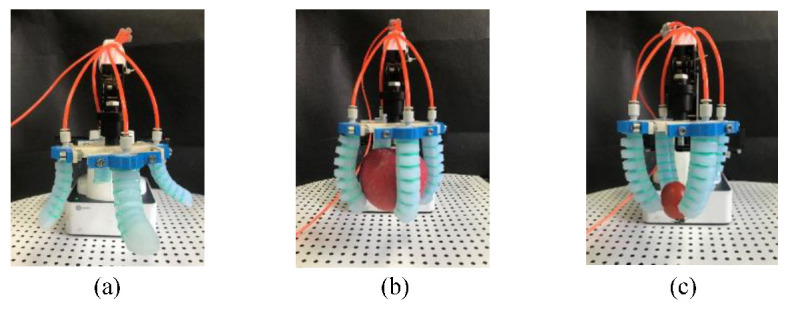
Soft manipulator grasping experiment. (**a**) Bending outward. (**b**) Enveloping grasping. (**c**) Fingertip grasping.

Due to the flexibility of its material, the soft manipulator could adapt to the contours of objects with different shapes. Moreover, even under excess grasping force, the soft finger can still avoid damaging the grasped object. Thus, the test shown in Figure 17 demonstrates the grasping performance of the soft manipulator that can grasp objects of different sizes and shapes. When grasping a larger object, a vacuum generator can be used to generate negative pressure first to make each soft actuator bend outwards (Figure 18a) to adapt to the size of the grasping object.

Enveloping grasping is to wrap the entire object with a large contact area with the object, and a greater grasping force is generated through the bending moment, which is suitable for larger objects such as apples, pears, tomatoes, and mangoes, as shown in Figure 17b,c. It can be seen that the soft manipulator can fit the various surfaces well, realize stable grasping, and does not damage the fragile grasped object.

The quality of various fruits and the selected pressure for each fruit are shown in Table 4. As the quality of the object increases, the required driving pressure also increases. When grasping mango, the driving pressure reaches the maximum, and it can be seen that the maximum load enveloped by the soft manipulator is about 5.8 N.

Fingertip grasping of an object depends on the friction generated by the contact force between the fingertip of the soft actuator and the surface of the object being grasped. Fingertip grasping only needs a small contact area, which is mainly used for small fruits such as cherry tomatoes and lychees. In this experiment, cherry tomatoes with a mass of 15.3 g were selected as the grasped target. When the driving pressure is 10 kPa, the cherry tomatoes can be successfully grasped, as shown in Figure 18c. Although fingertip grasping can achieve nondestructive grasping of small objects, the stability is insufficient, and the grasped object can easily fall off. To solve this problem, the contact area and friction coefficient could be tuned to improve grasp stability.

Comparing the two grasping methods, the envelope grasping can automatically adapt to the shape and size of the object being grasped and can realize the grasping of larger objects. Fingertip grasping can be used to grasp small objects. Since the anti-interference ability of grasping is weak, the stability of grasping can be achieved by appropriately increasing its rigidity. Reliable grasping for various objects with different shapes can be realized using the obtained soft manipulator by choosing suitable grasping types and adjusting structural parameters.

## 6. Conclusions

Due to its intrinsic advantages, the soft robot will soon play an important role in agricultural fields such as fruit harvesting and logistical sorting, including grasping or picking soft or fragile objects. This paper proposed a pneumatically actuated soft manipulator that can adjust its bending angle for fruit grasping. Analytical modeling of the soft manipulator was established to analyze the bending motion and output force of the soft actuator.

FEM analysis was conducted to explore the optimal structural parameters of pneumatic actuators, i.e., wall thickness of 2.5 mm, chamber gap of 3 mm, and restriction layer thickness of 2.5 mm. Furthermore, the simulation results show that the reinforced rings fixed on the soft actuator can increase its bending angle while maintaining its flexibility. Tests on bending angle and end-output force were conducted under 0–60 kPa air pressure, and the experimental results agree well with the FEM simulation and experiment results. The experiment indicates that the maximum output force of the soft manipulator is about 5.8 N. Two gripping manners of envelope grip and fingertip grip were demonstrated to grasp large and small fruits, respectively, exhibiting the tremendous application potential of the soft manipulator in fruit harvesting.

We have to admit that the grasping postures for various objects should be deeply discussed in future work.

## Figures and Tables

**Table 1:** Output force at the end of the soft actuator without constraint rings (F/N).

	Angle/(°)	0	15	30	45	60	75	90
Pressure/kPa								
10		0.07	/	/	/	/	/	/
20		0.24	0.15	0.08	/	/	/	/
30		0.46	0.36	0.29	0.21	0.11	/	/
40		0.72	0.61	0.53	0.45	0.36	0	/
50		1.08	0.95	0.84	0.73	0.64	0.52	0
60		1.36	1.22	1.09	0.95	0.84	0.71	0.55

**Table 2:** Output force at the end of the soft actuator with constraint rings (F/N).

	Angle/(°)	0	15	30	45	60	75	90
Pressure/kPa								
10		0.11	0.06	/	/	/	/	/
20		0.28	0.18	0.14	0.08	/	/	/
30		0.65	0.48	0.39	0.25	0.16	0.08	/
40		1.28	0.97	0.75	0.58	0.48	0.35	0.21
50		1.61	1.33	1.13	0.93	0.79	0.68	0.54
60		2.3	1.96	1.58	1.36	1.25	1.16	0.86

**Table 3:** The end-output force of fingertip grasping and envelope grasping.

Pressure/kPa	10	20	30	40	50	60
Fingertip grasping	0.41	0.79	1.29	1.51	1.85	2.13
Enveloping grasping	0.79	1.34	2.28	3.47	4.68	5.8

**Table 4:** The weight of the object to be grasped and the driving air pressure.

Fruit Varieties	Quality/g	Inflation Pressure/kPa
Apple	200.6	30
Tomato	282.9	35
Pear	389.1	45
Mango	580.3	60

## Data Availability

All test data mentioned in this paper will be made available on request to the corresponding author’s email with appropriate justification.
